# Industrial-Scale Production of Additive-Free Organic Salami Without Nitrite Curing Salt: Process Design and Technological Performance

**DOI:** 10.3390/foods15183301

**Published:** 2026-09-18

**Authors:** Sebastian Bühler, Angelika Ploeger, Hartmut Vogtmann

**Affiliations:** 1 Bäuerliche Erzeugergemeinschaft Schwäbisch Hall AG, 74523 Schwäbisch Hall, Germany; 2 Department of Organic Food Quality and Food Culture, University of Kassel, 37213 Witzenhausen, Germany

**Keywords:** organic salami, additive-free processing, nitrite-free curing, fermented sausage, industrial-scale production, microbiological safety, volatile N-nitrosamines, domestic heating

## Abstract

Replacing conventional curing agents in fermented sausages is technologically demanding, particularly in organic production, where nitrite curing salts are not permitted. This study validated an industrial-scale process for organic salami manufactured entirely without nitrite curing salt or other additives, and assessed volatile N-nitrosamine formation during domestic-style heating. Twenty production batches were monitored across two consecutive years under standardised industrial conditions. Samples from three batches were analysed for seven volatile N-nitrosamines before and after heating at 200 °C for 12 min and at 230 °C for 15 min by gas chromatography with thermal energy analysis. Fermentation was reproducible, yielding a mean final pH of 5.17 ± 0.18 with no significant year effect. Final water activity remained at or below 0.90 (mean 0.881), confirming dehydration as the decisive hurdle. Residual nitrate and nitrite remained low, and hygiene indicator counts were low; *Listeria monocytogenes*, *Salmonella* spp. and Shiga toxin-producing *Escherichia coli* were absent. All nitrosamines remained below the detection limit in unheated salami and after heating at 200 °C; small increases in N-nitrosodimethylamine, N-nitrosopiperidine and N-nitrosopyrrolidine occurred only under severe heating and remained toxicologically non-critical. For the product and conditions investigated, organic salami can therefore be produced safely and reproducibly at industrial scale without nitrite curing salt.

## 1. Introduction

The use of nitrite curing salt is a cornerstone of conventional fermented sausage manufacture, as it contributes to colour development, flavour stability, antioxidative protection, and microbial safety [1,2,3]. At the same time, nitrite is a key precursor for the formation of volatile N-nitrosamines (VNAs), a group of compounds that have raised long-standing toxicological concerns due to their carcinogenic potential demonstrated mainly in experimental studies and their classification by the International Agency for Research on Cancer (IARC) as probable human carcinogens [1,4,5,6,7]. As a result of this, consumer demand for clean-label meat products—that is, products formulated without additives requiring E-number declaration—has intensified in recent years [8], particularly in the organic sector, where the use of curing agents and other additives is strictly limited by regulation [9]. However, the elimination of nitrite curing salt represents a major technological challenge for fermented sausages. The renunciation of nitrite requires strict process control to compensate for its direct antimicrobial effects and its role in colour stabilisation and oxidative protection.

Several strategies have been proposed to reduce or replace conventional curing agents, including the use of vegetable-based nitrate sources and carefully designed fermentation and ripening regimes [10,11]. However, such approaches may introduce variability in residual nitrate and nitrite levels and can complicate colour formation. In nitrite-free fermented sausages, microbial stability depends primarily on rapid acidification during fermentation and the subsequent reduction in water activity during ripening. Consequently, precise control of temperature, relative humidity, airflow, mass loss, and pH development is essential to ensure product safety and technological consistency when nitrite is omitted [12]. Beyond production-related safety, the absence of nitrite also alters the potential for VNA formation. In fermented sausages, nitrite curing salts serve a dual function: they support microbial stability while simultaneously providing substrates for nitrosation reactions. Although the elimination of added nitrite reduces the availability of nitrosating agents, trace amounts of nitrate or nitrite may still originate from raw materials, spices, or microbial metabolism, leaving open the possibility of VNA formation under unfavorable conditions [3].

Thermal processing is a critical factor influencing VNA occurrence. While fermented sausages are typically consumed without heating, common consumer practices such as frying, grilling, or oven heating—particularly when used as pizza toppings—can expose products to surface temperatures sufficient to promote nitrosation reactions. Previous studies have demonstrated that VNA concentrations increase with rising temperatures and prolonged heating times, with cyclic nitrosamines such as N-nitrosopyrrolidine (NPYR) and N-nitrosopiperidine (NPIP) being especially affected [13]. Against this background, the present study aimed to develop and validate an industrial-scale process for producing organic salami without nitrite curing salts or other chemical additives, and to assess both technological and safety-related outcomes. The objectives were (i) to evaluate fermentation and ripening stability under standardized industrial conditions using chemical and microbiological indicators, and (ii) to investigate the potential formation of volatile N-nitrosamines in additive-free, nitrite-free organic salami before and after domestic-style heating under realistic and elevated temperature conditions. Biological nitrate reduction using plant-derived nitrate sources and nitrate-reducing starter cultures has been described previously; the specific contribution of the present work therefore does not lie in the curing principle as such, but in three aspects: the validation of a fully standardised process at genuine industrial scale across 20 batches and two consecutive production years, the demonstration of batch-to-batch reproducibility under commercial rather than pilot-plant conditions, and the assessment of volatile N-nitrosamine formation under realistic domestic heating, which has rarely been reported for nitrite-free fermented sausages.

## 2. The Technological Concept

### 2.1. Alternative Curing Concept Based on Biological Nitrate Reduction

The new processing concept was developed to manufacture organic fermented salami without the addition of nitrite curing salt (NPS) while ensuring microbiological safety, stable colour development, and technological reproducibility under industrial conditions [1,2,11]. The central technological feature of the process is the use of an alternative curing system that enables controlled colour formation in the absence of added nitrite, while microbiological stability is ensured by a hurdle-based process design culminating in a reduced water activity ($a_{w}$) at the end of ripening [11,14].

Instead of supplying nitrite via curing salt, the curing reaction is initiated by use of selected technological ingredients within the spice formulation that provide defined natural nitrate sources [2,3]. These components consist of dried vegetable- and spice-derived ingredients with a reproducible nitrate content [1,10]. Their quantitative composition must therefore be carefully adjusted to provide sufficient nitrate for colour formation while avoiding excessive residual nitrate or nitrite in the finished product [1,7]. The use of these ingredients is strictly functional and aims at enabling an alternative curing reaction rather than contributing to flavour.

Within the alternative curing concept, starter cultures play an important part and are applied with clearly differentiated and process-specific functions [15,16]. Lactic acid bacteria are used to initiate fermentation and to establish controlled acidification during the early processing stages [11,17]. Coagulase-negative staphylococci, in particular *Staphylococcus carnosus*, are applied primarily for their nitrate-reducing activity and thus play a central role in the alternative curing reaction in the absence of nitrite curing salt [1,15].

Within this system, naturally occurring nitrate derived from technologically functional vegetable and spice ingredients serves as the initial substrate [2]. During early fermentation and ripening, nitrate is enzymatically reduced to nitrite by nitrate reductase activity of *Staphylococcus carnosus* [11,15]. Subsequently, nitrite is further reduced to nitric oxide (NO) under the prevailing reductive conditions of the meat matrix [1,18]. This step is promoted by the presence of reducing agents, including L-ascorbic acid derived from acerola-containing ingredients, which facilitate electron transfer reactions and stabilise the reduced redox state [1,3].

The nitric oxide formed reacts with myoglobin to produce nitrosomyoglobin, resulting in stable cured colour formation [1,2]. During ripening, this pigment is further stabilised through dehydration and protein matrix consolidation [11]. The alternative curing reaction thus proceeds via a biologically mediated nitrate–nitrite–nitric oxide sequence, functionally equivalent to conventional nitrite-based curing, but without the addition of nitrite curing salt [1,3].

Quantitative optimisation of the natural nitrate input is critical within this system. Nitrate levels must be sufficiently high to enable reliable nitric oxide formation and colour development, yet sufficiently low to avoid accumulation of residual nitrite or nitrate in the finished product [1,7]. The controlled interaction between nitrate availability, nitrate-reducing staphylococci, reductive conditions, and process timing ensures that nitrite acts primarily as a transient intermediate rather than as a residual compound [2,3]. This biologically driven curing mechanism is the central element of the processing concept and forms the basis for achieving stable colour formation without NPS while maintaining low residual nitrite concentrations.

### 2.2. Conceptual Process Flow of the Alternative Curing Technology

**Figure 1:**
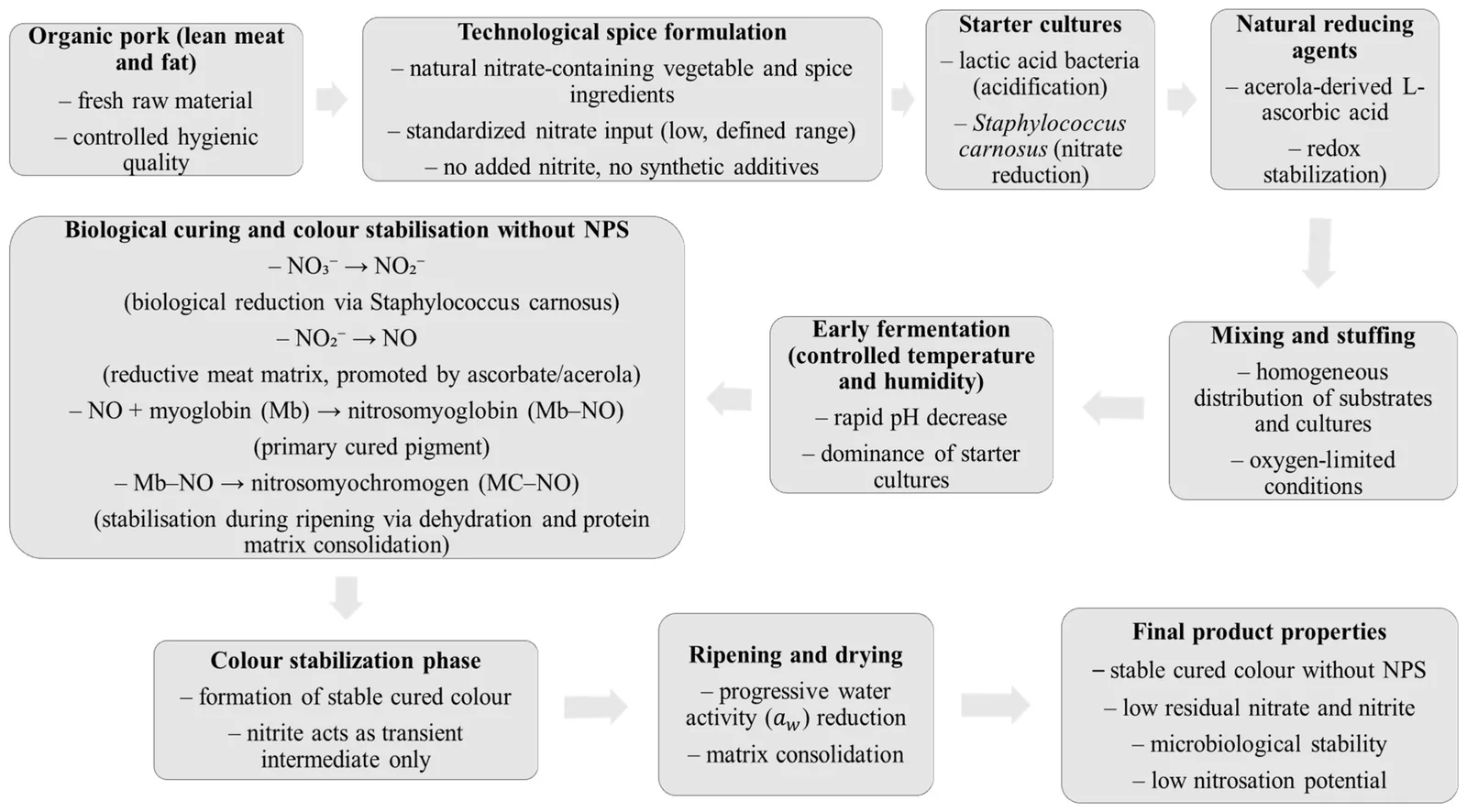
Process flow scheme of the alternative curing concept for additive-free organic salami production.

To provide an integrated overview of the processing concept, the complete production concept for additive-free organic salami is summarised in a conceptual process flow diagram (Figure 1). The flow sheet illustrates the sequence of unit operations from raw material selection to the final fermented product and highlights the key stages relevant for alternative curing, fermentation-driven stabilisation, and hurdle-based microbial control.

In contrast to conventional cured sausage manufacture, nitrite curing salt is not applied at any stage of the process. Instead, curing reactions are initiated biologically during early fermentation through the interaction of defined natural nitrate sources and nitrate-reducing starter cultures. The process flow therefore explicitly links raw material composition, microbial activity, and process conditions to colour development and product stabilisation.

Following comminution and mixing, fermentation and pre-ripening represent the critical phases in which acidification, nitrate reduction, and nitric oxide formation proceed in parallel. Subsequent ripening is characterised by progressive dehydration, which stabilises the cured colour and establishes the decisive hurdle for long-term microbiological stability via reduced water activity. Detailed process parameters, analytical verification, and sampling procedures are described in the Section 3.

The flow sheet illustrates the integration of raw materials, starter cultures, and process control steps enabling stable cured colour formation and microbiological safety in the absence of added nitrite. Natural nitrate sources derived from selected vegetable- and spice-based technological ingredients provide the substrate for a biologically mediated nitrate–nitrite–nitric oxide sequence driven by nitrate-reducing *Staphylococcus carnosus*. Nitrite acts only as a transient intermediate and does not accumulate in the final product. Early fermentation ensures rapid acidification, while progressive dehydration during ripening leads to reduced water activity ($a_{w}$), constituting the decisive hurdle for long-term microbiological stability. Dashed elements indicate key process control parameters (pH, mass loss, temperature, humidity) supporting reproducibility under industrial conditions.

## 3. Materials and Methods

### 3.1. Raw Materials and Formulation

Organic pork was obtained from the Farmers’ Producers Association Schwäbisch Hall (Bäuerliche Erzeugergemeinschaft Schwäbisch Hall AG, Schwäbisch Hall, Germany) and processed in accordance with EU organic production standards (Regulation (EU) 2018/848) [9]. All salami batches were manufactured at the company’s dry-sausage plant in Frankenheim (Meiningen district, Thuringia, Germany), located at approximately 800 m above sea level in the Rhön Biosphere Reserve. After deboning, meat cuts were sorted visually according to lean and fat content prior to comminution. Each batch comprised 156.05 kg of raw materials: 35.0 kg frozen pork back fat without rind (22.43%), 35.0 kg frozen pork trimmings of grade S I (22.43%), 80.0 kg desinewed pork trimmings (51.27%), 2.10 kg organic spice mixture (1.35%), 3.90 kg natural rock salt (2.50%) and 0.050 kg starter culture preparation (0.03%). The meat batter was filled into diffusion-permeable, peelable artificial casings (calibre 60–80 mm; stick length up to approximately 1.2 m) to support controlled moisture exchange during fermentation and ripening.

The organic spice mixture was added at 14 g/kg of batter. Its composition (% of the mixture) was: black pepper 14.29, red beet powder 21.43, dextrose 7.86, garlic powder 5.71, vegetable bouillon 5.71, vegetable seasoning preparation 7.14, vegetable powder preparation 7.14, parsnip powder 5.07, anise 5.00, fennel 5.00, coriander 3.57, ground acerola 2.86, allspice 2.86, beet sugar 1.43, sweet paprika 1.43, caraway 1.05, bay leaf 1.02, blue fenugreek 0.71 and maltodextrin 0.71. Nitrate was supplied exclusively by the vegetable components of this mixture, in particular red beet, parsnip and the vegetable powder preparation, which provided reproducible natural nitrate contents enabling biologically mediated curing in the absence of nitrite curing salt. Their inclusion level was adjusted to provide sufficient nitrate for colour development while avoiding excessive residual nitrate or nitrite in the final product.

Reducing capacity was provided exclusively via natural sources, in particular ground acerola (Malpighia emarginata D.C.), a fruit reported to contain between 500 and 4500 mg ascorbic acid per 100 g [19], which supports reductive conditions within the meat matrix and promotes the conversion of nitrite to nitric oxide during early fermentation and pre-ripening. Dextrose and beet sugar served as fermentable substrates for controlled acidification, dextrose being metabolised first as a readily available monosaccharide.

Two commercial starter culture preparations were used, both supplied by Frutarom Savory Solutions (today NovaTaste), Salzburg, Austria: a mild, fast-acidifying culture containing *Lactobacillus sakei*, and the preparation SM96, containing predominantly *Staphylococcus carnosus*. They were added together with the spice mixture during the second cutting step, at a combined level of 50 g per cutter filling, corresponding to 0.32 g/kg of batter. *L. sakei* provided homofermentative acidification and acted as a bioprotective culture, while *S. carnosus* provided the nitrate reductase activity required for the biological nitrate-to-nitrite conversion, together with catalase activity and aroma formation.

No nitrite curing salt, nitrate curing salt, synthetic antioxidants, or other additives requiring declaration by E-numbers were used at any stage of production. The formulation and the processing concept were developed and documented in the framework of the doctoral thesis of the corresponding author [20].

### 3.2. Comminution, Stuffing and Ripening

Frozen back fat and frozen pork trimmings were cut into homogeneous blocks of 10 × 5 × 5 cm using a frozen-block cutter (MAGURIT Gefrierschneider GmbH, Remscheid, Germany). The non-frozen portion of the trimmings was desinewed on a mechanical desinewing unit (SEPAmatic, Overath, Germany), which simultaneously reduced the particle size to approximately 2 mm; owing to the increased specific surface area, this material was processed without delay. Meat components were weighed on a floor scale and recorded in the plant management system, while spices, salt and cultures were weighed separately in a dedicated mixing room.

Comminution was performed in a 340 L raw-sausage bowl cutter equipped with six blades and operated by an automated programmable control system (Seydelmann, Stuttgart, Germany). Frozen fat was loaded first at an initial temperature of approximately −12 to −10 °C and cut for 45 s at a blade frequency of 994 rpm with a bowl speed of 7.5 rpm. The spice mixture and the starter cultures were then added and distributed for a further 75 s under the same settings, after which the frozen and desinewed pork trimmings were incorporated. Blade frequency, bowl speed, duration and temperature were controlled automatically for each processing step. The batter was stuffed at approximately −2.6 °C.

Ripening was separated spatially into a pre-ripening and a post-ripening stage. Pre-ripening lasted approximately one week and followed a standardised 16-phase programme. Temperature, relative humidity and air velocity were regulated by a programmable climate control system and verified regularly using manual instruments. The programme started at 14 °C and 94% relative humidity (RH) for 8 h, followed by a warm phase at 24 °C and 94% RH for 24 h and a further 12 h at 23 °C and 92% RH, which provided conditions for nitrate reduction and acidification. Relative humidity was then lowered to 51% at 18 °C for 3 h prior to the first smoking phase. Five cold-smoking phases were applied at 18, 17, 15, 14 and 13 °C, each at 51% RH and lasting 1–2 h; smoking temperature was always kept below the surface temperature of the preceding ripening phase to prevent surface condensation. Between smoking phases, ripening continued at 20 °C/84–90% RH (2 and 16 h), 18 °C/86% RH (18 h), 18 °C/84% RH (24 h), 16 °C/82% RH (24 h) and 15 °C/80% RH (24 h), producing a gradual and controlled drying trajectory that avoided case hardening.

Cold smoke was generated by a friction smoke generator in which beech wood billets were abraded by a rotating drum. Post-ripening was subsequently carried out under constant conditions at 10 °C (range 9–11 °C) and 78% RH (range 74–80%). Process control points were set at 7-day intervals, at which mass loss and pH were determined on a defined reference rack per batch; typical mass losses were approximately 20% and 25% at the first and second control points. Ripening was terminated when the target values were reached: 28% mass loss, water activity below 0.93 and pH 5.1–5.2.

### 3.3. Chemical and Physical Analyses

The pH was measured at defined stages of the ripening process, including an early ripening stage at approximately day 7, coinciding with the minimum pH reached during fermentation, and at the end of ripening after completion of drying and stabilisation, using a calibrated penetration electrode according to ISO 2917 [21]; in-process pH was additionally verified with a portable penetration pH meter (pH-STAR; Matthäus GmbH & Co. KG, Eckelsheim, Germany). The early measurement reflects the phase of maximum lactic acid bacteria activity and early microbiological stabilisation, whereas the final measurement represents the stable endpoint of the process after achieving the target degree of dehydration. In addition to these two endpoints, pH and mass loss were recorded at the routine process control points at approximately 7-day intervals throughout post-ripening, allowing the intermediate trajectory to be followed for each batch. Residual nitrate and nitrite contents were determined photometrically by an accredited laboratory following EN/DIN 12014-7 [22]. Water activity (aw) was measured in the finished products at the end of ripening using a chilled-mirror dewpoint water activity meter (AQUALAB 4TE; Addium GmbH, Munich, Germany) at 25 °C, calibrated against certified saturated salt standards prior to measurement. Water activity was recorded to two decimal places.

Volatile N-nitrosamines (VNAs) were quantified by an accredited ISO/IEC 17025 [23] testing laboratory (Normec Institut METAKOM Kompetenzzentrum für Lebensmittelsicherheit GmbH & Co. KG, Feuchtwangen, Germany) using gas chromatography coupled with a thermal energy analyzer (GC–TEA) in accordance with the official German method ASU L 00.00-17 [24]. The analytical panel comprised seven compounds relevant to heat-induced nitrosamine formation in meat products, namely N-nitrosodimethylamine (NDMA), N-nitrosodiethylamine (NDEA), N-nitrosodipropylamine (NDPA), N-nitrosodibutylamine (NDBA), N-nitrosomorpholine (NMOR), N-nitrosopiperidine (NPIP), and N-nitrosopyrrolidine (NPYR). For all analytes, the limit of detection (LOD) was <0.5 µg/kg.

### 3.4. Microbiological Analyses

The microbiological status of the finished products was assessed by determining total viable counts [25] as well as relevant hygiene indicators, pathogens, and spoilage organisms using ISO reference methods: *Escherichia coli* [26], coagulase-positive staphylococci [27], *Enterobacteriaceae* [28], *Salmonella* spp. [29], and *Listeria monocytogenes* [30,31]. Compliance with microbiological quality and safety criteria was evaluated according to the guideline and warning values of the Deutsche Gesellschaft für Hygiene und Mikrobiologie [32] for sliced fermented sausages (salami) at the trade level.

### 3.5. Data Handling and Statistical Analysis

Chemical, microbiological, and nitrosamine data were compiled for each production batch. For the industrial production study, data were evaluated on a batch basis across two consecutive production years. The experimental unit was the individual production batch; each of the 20 batches (10 per production year) contributed one value per parameter, and results are reported as mean ± standard deviation. Prior to statistical analysis, data were tested for normality (Anderson–Darling test) and homogeneity of variances (Levene test); given the limited number of batches per year, these test outcomes were interpreted with caution. Year effects were assessed by one-way analysis of variance (ANOVA), with differences considered statistically significant at *p* < 0.05. For the investigation of volatile N-nitrosamines, all analyses were performed in triplicate, corresponding to three independent production batches. Mean values and standard deviations (SD) were calculated for each heating condition. Differences among thermal treatments were assessed by analysis of variance (ANOVA), with *p* < 0.05 considered statistically significant, applying the same significance level as above. Concentrations below the analytical detection limit (LOD; <0.5 µg/kg) were treated as left-censored data and reported as “<0.5 µg/kg”. The relationship between residual nitrate and nitrite was examined by Pearson correlation, with the correlation coefficient (r) and the corresponding *p*-value reported in the Results. This approach avoids numerical substitution and prevents distortion of the underlying data distribution, which is consistent with recommended practice for censoring in chemical residue analysis. All statistical analyses were performed using Minitab 17 (Minitab Inc., State College, PA, USA).

## 4. Industrial-Scale Salami Production

### 4.1. Experimental Design

The industrial production process was designed to provide appropriate temporal and environmental conditions for the alternative curing reaction to proceed in a controlled manner. Processing followed a standardised sequence including cutting and mixing, filling, hanging, and transfer to climate-controlled ripening areas. Sausages were filled into the casings described in Section 3.1 to enable uniform moisture exchange. Product spacing on smoke sticks and airflow conditions were standardised to ensure homogeneous process conditions across all units. Fermentation and ripening were conducted using a programmed climate regime controlling temperature, relative humidity, airflow, and smoking. During the early stages, fermentation, nitrate reduction, and colour formation proceed in parallel. As ripening progresses, dehydration becomes increasingly dominant. In contrast to nitrite-based preservation, microbiological stability in the present concept is not achieved through curing agents but through the deliberate application of the hurdle principle, with several supportive hurdles acting sequentially.

During the early stages of fermentation, rapid acidification driven by lactic acid bacteria leads to a decrease in pH, which limits the growth of undesirable microorganisms and supports safe process progression. In the present process, fermentation was designed to achieve a temporary pH reduction to values below 5.0, which constitutes a relevant early antimicrobial hurdle in the absence of nitrite curing salt. At the same time, the onset of dehydration was used as a controlled and documented process indicator: the early ripening phase was managed to reach approximately 10% mass loss (for ≤80 mm calibre products) at the point when pH values fell below 5.0. This combined pH–dehydration state represents a critical early-stage hurdle constellation that stabilises the product during the transition from fermentation to ripening. Process progression was documented by monitoring mass loss on a defined reference rack per batch, enabling verification of a reproducible drying trajectory. In parallel, the dominance of starter cultures, including *Staphylococcus carnosus*, contributes to competitive exclusion of spoilage and pathogenic bacteria and stabilises the microbial ecosystem during curing and early ripening. In this context, starter cultures act as protective cultures by rapidly establishing a desirable microbial consortium and suppressing the outgrowth of competing flora during the initial phase when the antimicrobial contribution of nitrite is absent. The protective effect results from rapid fermentation-driven acidification, nutrient competition, and the creation of an environment favouring the starter microbiota, thereby supporting hygienic process stability prior to completion of dehydration-driven stabilisation.

As ripening proceeds, a moderate increase in pH to approximately 5.2 may occur, which is a typical phenomenon in fermented sausages and is associated with proteolytic activity and matrix stabilisation. At this stage, microbiological stability is increasingly governed by physical rather than chemical hurdles and dehydration becomes increasingly dominant as a safety factor. Within this hurdle-based design, acidification and starter culture dominance act as preparatory barriers, while the decisive and final hurdle ensuring long-term microbiological stability is the reduction in water activity (aw) achieved during ripening. The ripening process was therefore designed to reach a low aw-value at the end of processing, typically ≤0.90, which represents a threshold below which the growth of relevant pathogenic microorganisms is inhibited. The end state of stabilisation was controlled via the drying endpoint, with ripening continued until a total mass loss of approximately 28% was achieved, which corresponds to the targeted low aw-range required for sustained microbial stability. Achieving an aw-value in this range ensures that pathogens such as *Listeria monocytogenes*, *Salmonella* spp., and *Escherichia coli* are unable to grow, thereby providing sustained microbiological stability despite the absence of nitrite curing salt (NPS). The final aw reduction thus constitutes the key safety barrier of the processing concept, while the preceding hurdles compensate for the missing antimicrobial effect of nitrite during earlier processing stages. Overall, the hurdle sequence can be summarised chronologically as follows: (i) immediate dominance of protective starter cultures, (ii) rapid acidification to pH < 5.0 during fermentation, (iii) controlled early dehydration documented by approximately 10% mass loss as fermentation transitions into ripening, and (iv) final dehydration to a low aw-value (typically ≤ 0.90), achieved at approximately 28% total mass loss, which provides long-term microbiological stability in the finished product.

### 4.2. Experiment and Sampling

#### 4.2.1. Production Design and Processing Conditions

Organic fermented salami was manufactured without the use of nitrite curing salt or synthetic additives under industrial production conditions using the alternative curing concept described in Section 2. Production trials were conducted in a commercial processing facility and followed a programmed fermentation and ripening regime established during prior process development and validation.

Production was carried out over two consecutive years under identical and predefined processing conditions to evaluate process robustness and reproducibility. In each year, ten independent production batches were manufactured and sampled at approximately five-week intervals, resulting in a total of 20 full-scale batches across both years.

After comminution and mixing of raw materials, technological ingredients, and starter cultures, the meat batter was filled into the casings described in Section 3.1 and subjected to a controlled, multi-stage fermentation and ripening program comprising:

Fermentation/pre-ripening phase: Conducted at elevated temperatures of approximately 24–26 °C and high relative humidity (>90%) to promote rapid starter culture activity. During this phase, lactic acid bacteria ensured controlled acidification, while nitrate-reducing *Staphylococcus carnosus* facilitated biological nitrate reduction. The pH decreased from initial values of approximately 5.6–5.8 to minimum values around 5.0–5.2, typically reached within the first 7–10 days.

Ripening phase: Following fermentation, temperature and relative humidity were progressively reduced over several weeks to promote gradual dehydration and structural consolidation of the sausage matrix. Dehydration was monitored by mass loss and aimed at achieving a low final water activity; the process was designed to reach final $a_{w}$-values typically ≤0.90, constituting the decisive hurdle for long-term microbiological stability.

All relevant processing parameters (temperature profiles, relative humidity, air circulation, and process duration) were identical for all batches across both production years and followed fixed, pre-defined setpoints.

#### 4.2.2. Sampling Strategy

For chemical and microbiological evaluation, five finished salami sticks (approximately 2 kg each) were randomly selected from each batch. From the central section of each salami, ten central slices were collected and pooled to form one composite sample per batch. This sampling strategy was chosen to minimise within-product variability and to obtain representative samples of the finished products.

#### 4.2.3. Nitrosamine Experiment and Thermal Treatment

The investigation of volatile N-nitrosamine (VNA) formation was conducted as a targeted follow-up experiment during the second production year only. For this purpose, three independent production batches were selected. From each batch, salami slices were collected from the central section of the finished product and prepared for nitrosamine analysis. The sausages were sliced with a commercial slicer to a thickness of approximately 1.6 mm, corresponding to the producer’s standard specification for sliced product.

Samples were analysed in the unheated state as well as after defined thermal treatments simulating domestic preparation and high-temperature stress conditions. Heating was performed on a baking tray in a precisely regulated convection oven under controlled conditions; the stated temperatures refer to the oven set temperature; the core temperature of the slices was not recorded, although the thin and uniform slice geometry means that the samples equilibrated rapidly towards the oven temperature. The lower condition corresponds to the heating instructions commonly recommended for frozen pizza, whereas the higher condition represents an exaggerated scenario in which the recommended range is exceeded. After heating, samples were cooled to room temperature and homogenised prior to chemical analysis.

The applied treatments were:

(i) unheated (control);
(ii) heating at 200 °C for 12 min;
(iii) heating at 230 °C for 15 min.

## 5. Results

### 5.1. pH Development

From a technological perspective, the pH value in additive-free fermented salami fulfils a dual function. On the one hand, acidification during fermentation contributes to microbiological stability; on the other hand, excessive acidification may negatively affect sensory properties by promoting an overly sour taste. For fermented sausages without nitrite curing salt, a target pH range of approximately 5.0 to 5.3 is generally considered favourable, contributing to microbial inhibition while maintaining balanced flavour development. It should be emphasised that acidification within this range does not by itself ensure inhibition of *Listeria monocytogenes* or other acid-tolerant pathogens; in the present concept it acts as an early, supportive hurdle, whereas the decisive barrier is the reduction in water activity achieved during ripening [12,14]. Growth of *L. monocytogenes* requires a water activity of approximately 0.92 or higher; the final values obtained here (0.87–0.90; mean 0.881) are therefore below the threshold permitting proliferation of this organism, although a low water activity restricts growth rather than inactivating cells that may already be present.

**Figure 2:**
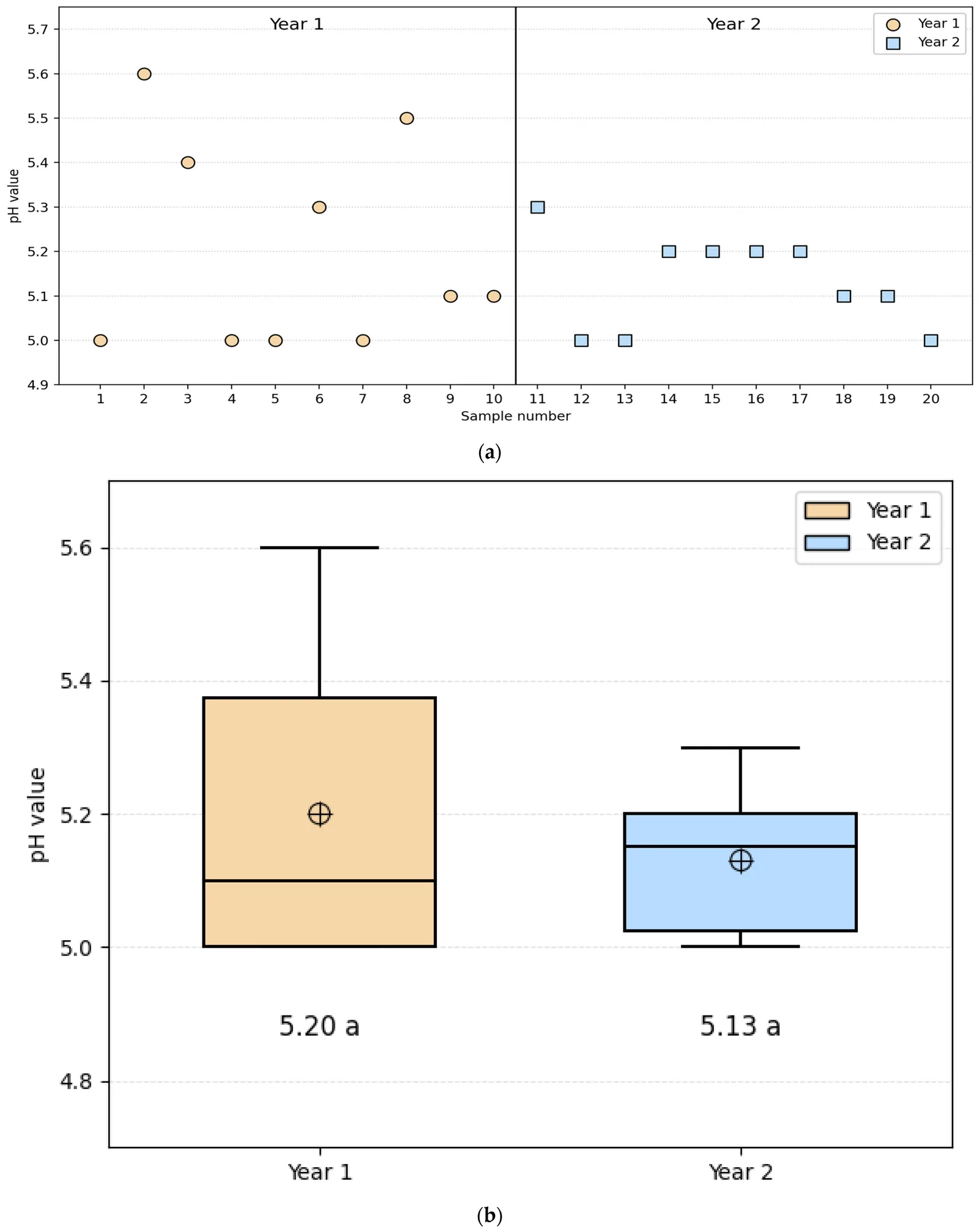
(**a**) Individual final pH values of additive-free organic salami from two consecutive production years. Each symbol represents one production batch (*n* = 20; circles, year 1; squares, year 2); the vertical line separates year 1 and year 2. Mean values were 5.20 ± 0.23 (year 1) and 5.13 ± 0.11 (year 2); the difference between years was not significant (ANOVA, *p* > 0.05). (**b**) Distribution of final pH values in additive-free organic salami by production year. Data are shown as boxplots (median, interquartile range, minimum and maximum), with mean values indicated by ⊕. Identical letters indicate no significant differences between years (ANOVA, *p* > 0.05).

The final product pH values of additive-free organic salami are shown in Figure 2. Figure 2a illustrates the individual pH values of all samples from the first and second production years, while Figure 2b summarises the distributions by year using boxplots. In both production years, final pH values clustered around 5.2, with only moderate sample-to-sample variation. In year 1, pH values ranged from 5.0 to 5.6, with a mean of 5.20 ± 0.23. In year 2, the range was narrower, extending from 5.0 to 5.3, with a mean of 5.13 ± 0.11. Across all 20 batches, the mean final pH was 5.17 ± 0.18. Thus, while the average pH values were comparable between years, the variability was reduced in the second production year. This illustrates that pH values did not fall below the critical threshold of pH 5.0. In several samples from year 1 (three of ten), pH values slightly exceeded the upper end of the optimal range. However, this did not result in reduced microbiological stability, as confirmed by the microbiological analyses of the corresponding samples. In year 2, the spread of pH values was smaller (SD 0.11 versus 0.23 in year 1), indicating improved control of fermentation and ripening conditions.

The boxplot representation (Figure 2b) confirms comparable medians and narrow interquartile ranges for both years. The pH range observed in year 1 (5.0–5.6) is comparatively wide for a fermented sausage; this reflects the variability inherent to full-scale industrial production, and the spread was reduced in year 2 as process control was refined. Beyond the two endpoints reported above, the intermediate trajectory followed a consistent pattern across batches: after approximately 7 days of pre-ripening the pH had fallen below 5.0, corresponding to about 10% mass loss; it then rose gradually during post-ripening, reaching 5.1–5.2 at the end of ripening at approximately 28% mass loss. This gradual increase is typical of fermented sausages and is associated with proteolytic activity and matrix stabilisation. Statistical analysis revealed no significant difference in final pH values between production years (*p* > 0.05). The reduced variability observed in the second year is consistent with the refined process control implemented after the first production year, although this difference was not statistically significant. Overall, the results demonstrate stable and reproducible fermentation behaviour under industrial conditions and confirm that the alternative curing concept allows reliable pH management without the use of nitrite curing salt.

### 5.2. Water Activity (aw)

Water activity (aw) constitutes the decisive hurdle for long-term microbiological stability in additive-free, nitrite-free salami, since the antimicrobial contribution of nitrite is absent. The final aw was therefore monitored as the key safety-related endpoint of the ripening process, with a target threshold of ≤0.90 below which the growth of relevant pathogenic microorganisms is inhibited.

**Figure 3:**
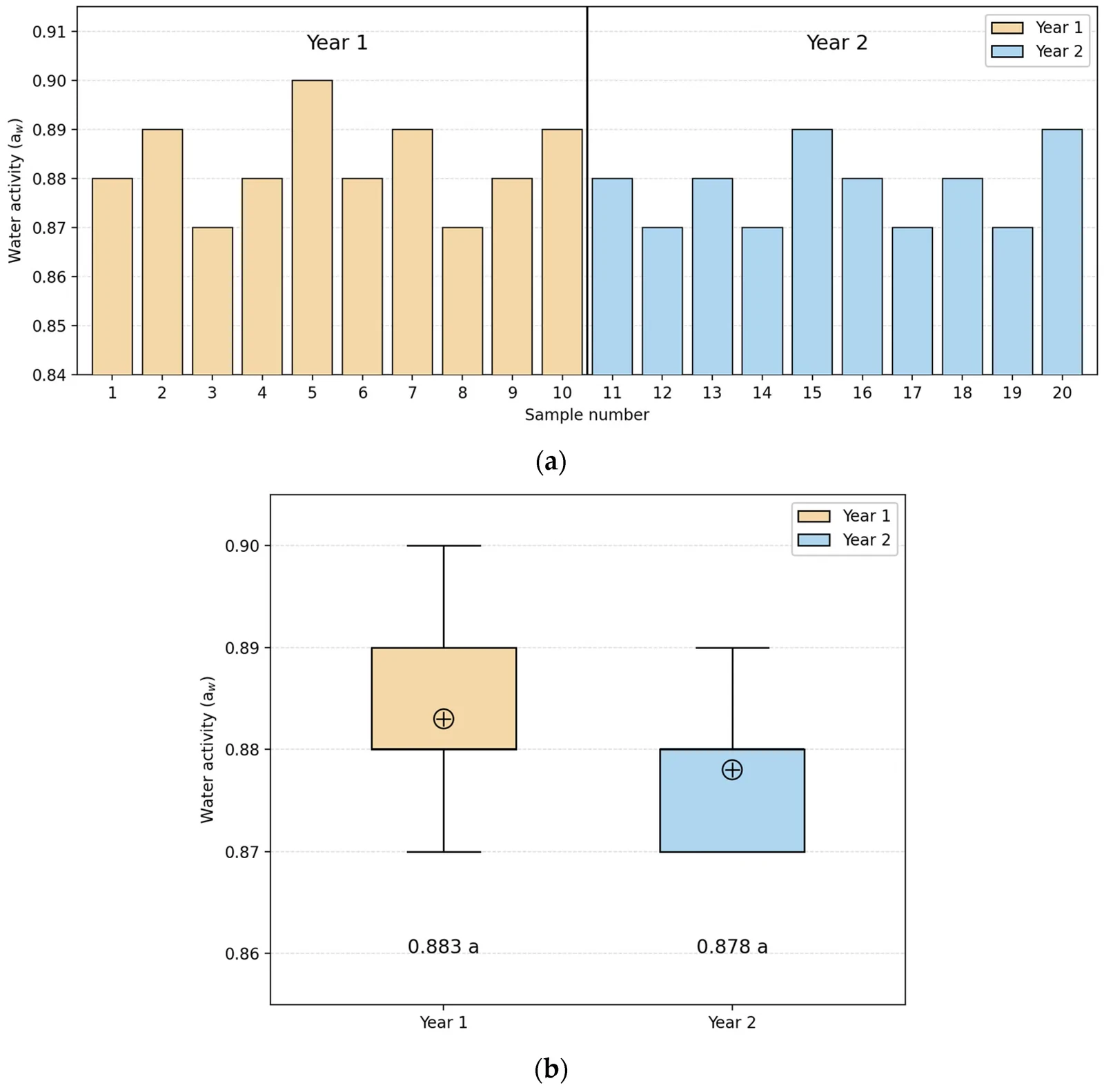
(**a**) Individual final water activity (aw) values of additive-free organic salami from two consecutive production years. Bars represent single samples; the vertical line separates year 1 and year 2. (**b**) Distribution of final water activity (aw) in additive-free organic salami by production year. Data are shown as boxplots (median, interquartile range, minimum and maximum), with mean values indicated by ⊕. Identical letters indicate no significant differences between years (ANOVA, *p* > 0.05).

The final water activity values of additive-free organic salami are shown in Figure 3. In both production years, final aw values remained at or below the target threshold of 0.90. In year 1, aw values ranged from 0.87 to 0.90, with a mean of 0.883. In year 2, the range was narrower, extending from 0.87 to 0.89, with a mean of 0.878. The overall mean across both years was 0.881 (range 0.87–0.90, *n* = 20), and the majority of all batches reached final aw values of 0.87 or 0.88. As water activity was recorded to two decimal places, the observed range is reported here as the measure of dispersion. Statistical analysis revealed no significant difference in final aw values between production years (*p* > 0.05); nevertheless, the lower maximum and reduced spread observed in the second year are consistent with the improved process control already reflected in the pH and residual nitrate data. These results confirm that the targeted reduction in water activity was reliably achieved under industrial conditions, establishing the decisive hurdle required for sustained microbiological stability in the absence of nitrite curing salt.

### 5.3. Nitrate and Nitrite Concentrations

**Figure 4:**
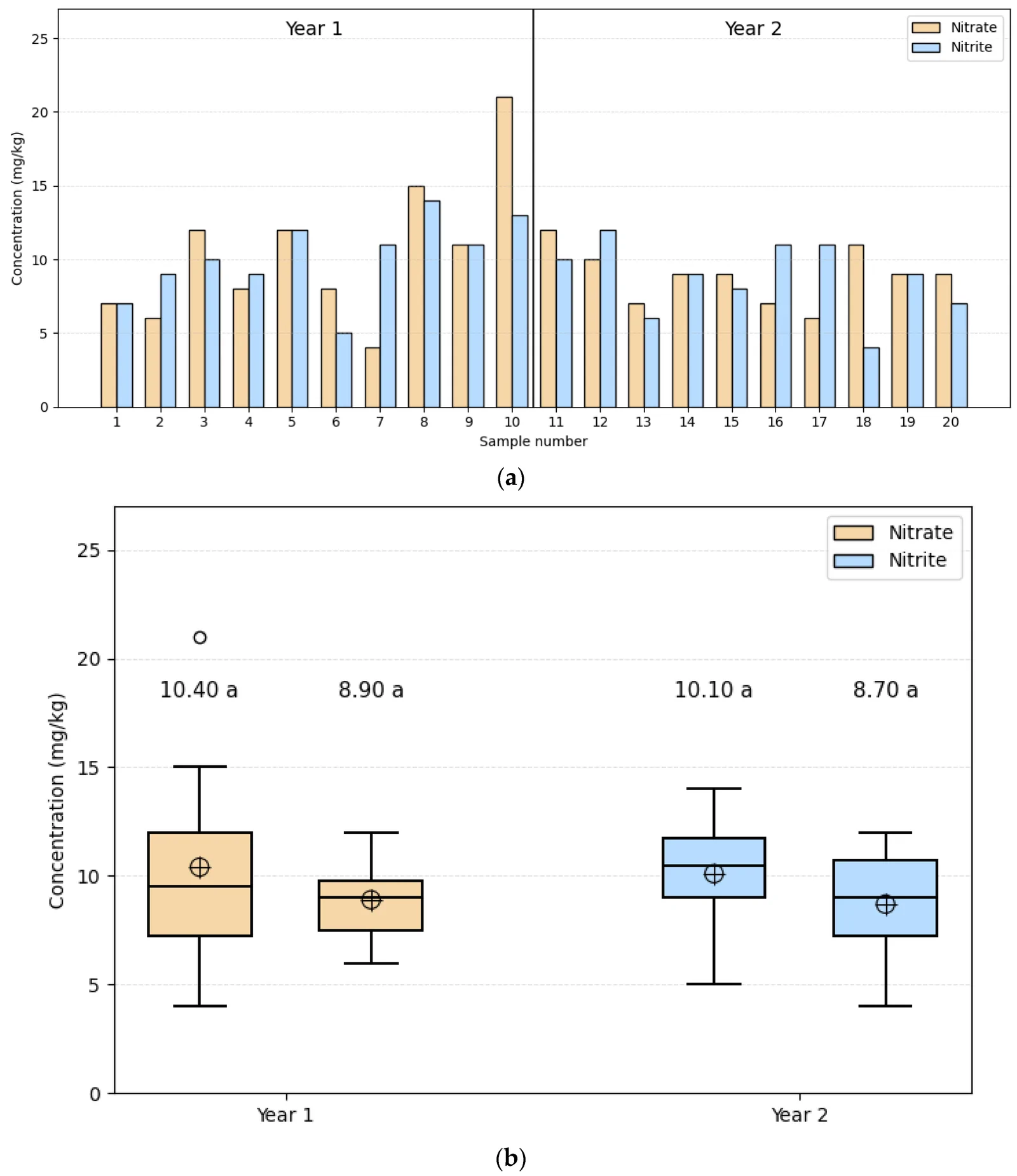
(**a**) Individual nitrate (NO_3_^−^) and nitrite (NO_2_^−^) concentrations in additive-free organic salami from two consecutive production years. Bars represent single samples; the vertical line separates year 1 and year 2. (**b**) Distribution of nitrate (NO_3_^−^) and nitrite (NO_2_^−^) concentrations in additive-free organic salami by production year. Data are shown as boxplots (median, interquartile range, minimum and maximum), with mean values indicated by ⊕. Identical letters indicate no significant differences between years (*p* > 0.05).

The nitrate and nitrite concentrations of additive-free organic salami produced during the first and second production years did not exhibit a uniform pattern across individual samples (Figure 4a). This variability is not unexpected, as the nitrate content of naturally derived vegetable- and spice-based technological ingredients is subject to batch-related fluctuations. In addition, minor variations in process conditions during the pre-ripening phase, in which nitrate serves as the initial substrate for redox-driven curing reactions, may influence residual nitrate and nitrite levels. Factors such as local airflow, temperature distribution, and the spatial positioning of sausages within the ripening chamber can contribute to such differences under industrial conditions.

Across all samples, nitrate concentrations ranged from 4 to 21 mg/kg. The overall mean nitrate concentration across both production years was 9.65 ± 3.73 mg/kg salami, which can be considered low. No regulatory maximum limit for nitrate in fermented sausages is currently defined. When evaluated by production year, nitrate concentrations in year 1 ranged from 4 to 21 mg/kg with a mean of 10.40 ± 4.97 mg/kg, whereas in year 2 values ranged from 6 to 12 mg/kg with a mean of 8.90 ± 1.85 mg/kg. The boxplot representation (Figure 4b) highlights one higher nitrate value in year 1 (21 mg/kg), which is classified as an outlier. With only ten values per year, tests of distribution have limited power, and a single deviating value corresponds to 10% of the annual dataset; such a value may indicate an atypical sausage, a deviation during sampling, or analytical variability. It was retained in the dataset because no procedural error could be identified. Nitrite concentrations were similarly low in both production years. In year 1, nitrite values ranged from 5 to 14 mg/kg with a mean concentration of 10.10 ± 2.73 mg/kg, while in year 2 values ranged from 4 to 12 mg/kg with a mean of 8.70 ± 2.50 mg/kg. Across all 20 batches, the mean residual nitrite concentration was 9.40 ± 2.64 mg/kg. Both the individual-value plot and the boxplot representation demonstrate comparable distributions between years for nitrite, with no indication of systematic accumulation.

Statistical analysis revealed no significant effect of production year on either nitrate or nitrite concentrations (*p* > 0.05). Although a slight reduction in mean nitrate and nitrite concentrations was observed in the second production year, this difference cannot be considered statistically significant. Nevertheless, the tendency toward lower residual concentrations in year 2 coincides with improved standardisation of process parameters during the pre-ripening phase, including enhanced electronic control of temperature, relative humidity, and airflow. These adjustments were implemented based on observations made during the first production year.

**Figure 5:**
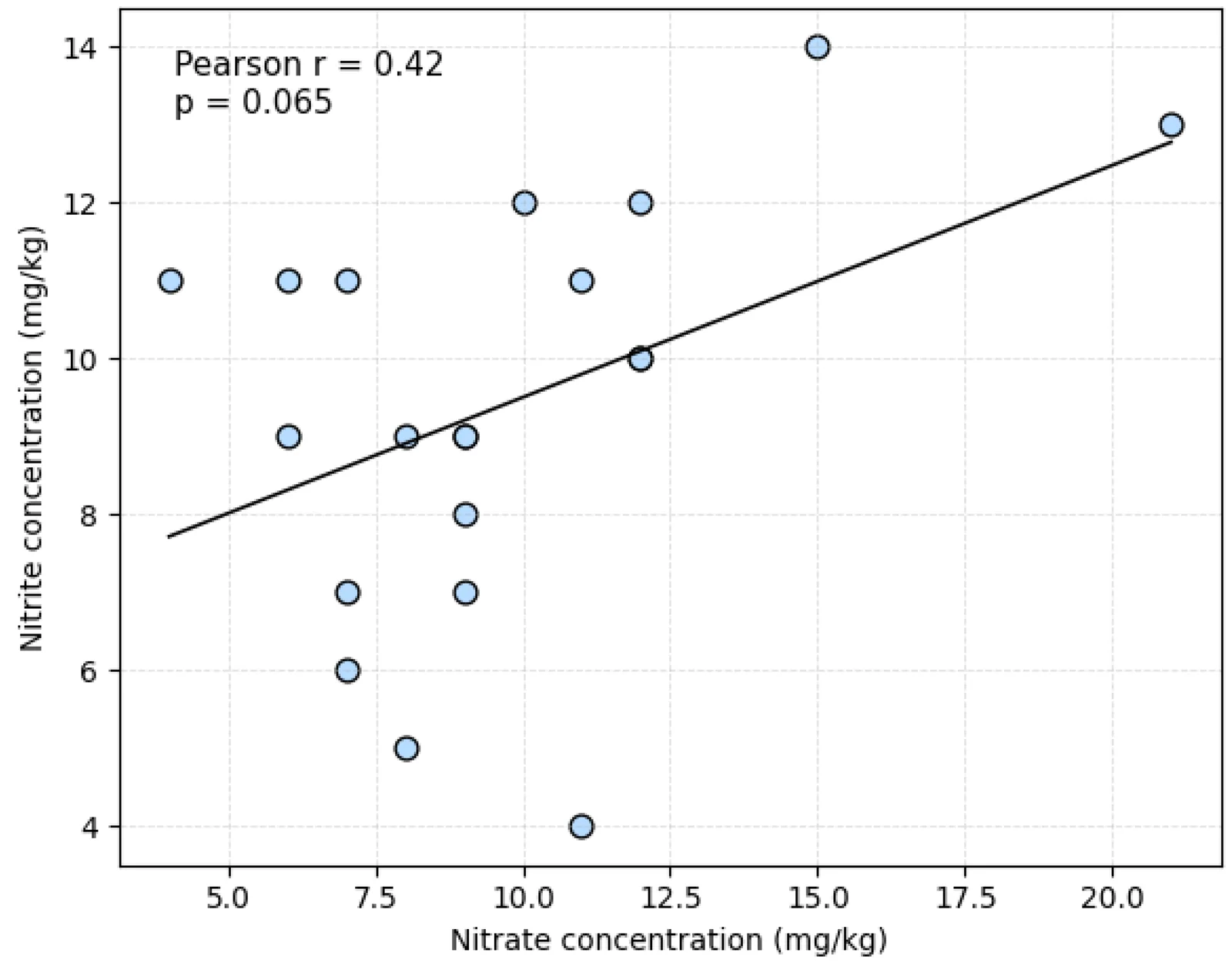
Relationship between residual nitrate (NO_3_^−^) and nitrite (NO_2_^−^) concentrations in additive-free organic salami. Each data point represents one individual sample. No significant correlation was observed between nitrate and nitrite concentrations (Pearson correlation, *p* > 0.05).

Based on the reaction sequence governing alternative curing:

(NO_3_^−^ ⇌ NO_2_^−^ ⇌ HNO_2_ → NO → Mb–NO → MC–NO),

a correlation between residual nitrate and nitrite concentrations might be expected. However, statistical evaluation did not reveal a significant relationship between these parameters (Figure 5). This finding can be explained by the fact that the concentrations of individual intermediates within the curing pathway are influenced by different, partially independent process parameters. Temperature, oxygen availability, pH, water activity, and microbial activity vary temporally and spatially during fermentation and pre-ripening, resulting in different reaction extents and rates for nitrate reduction, nitrite formation, and subsequent nitric oxide generation. Consequently, transient accumulation of nitrite does not necessarily correspond to the residual nitrate concentration measured at the end of ripening.

From a toxicological perspective, the observed nitrate and nitrite concentrations are uncritical. The World Health Organization defines an acceptable daily intake (ADI) for nitrate of 3.7 mg/kg body weight, corresponding to 259 mg/day for a 70 kg adult. Even with a daily consumption of 250 g salami, nitrate intake from the present product would amount to approximately 2.5 mg/day and is therefore negligible compared with other dietary nitrate sources such as vegetables or drinking water [33]. For nitrite, EU legislation specifies maximum levels of 50–175 mg/kg depending on product category. With mean nitrite concentrations below 9 mg/kg, the additive-free salami produced using the alternative curing concept remains far below these regulatory thresholds. Overall, both nitrate and nitrite levels in the present products can be regarded as safe from a consumer health perspective.

### 5.4. Microbiological Analysis

The microbiological status of the additive-free organic salami is summarised in Table 1. All analysed samples (*n* = 20) exhibited a favourable microbiological profile. *Escherichia coli* counts remained below the detection limit of 10 CFU/g in all samples. *Enterobacteriaceae* were detected only at low levels, ranging from 10 to a maximum of 200 CFU/g. Coagulase-positive staphylococci were present at very low concentrations, with counts between 1 and 20 CFU/g. No pathogenic microorganisms were detected. *Listeria monocytogenes* was not detected by either quantitative enumeration or qualitative testing of 25 g test portions. Likewise, *Salmonella* spp. were absent in all samples analysed. PCR screening for Shiga toxin-producing *Escherichia coli* (STEC/VTEC), targeting the *stx1* and *stx2* genes, yielded negative results in all cases. All detected microbial counts were well below the guideline and warning values established for fermented sausages. No differences between production years were observed. These findings indicate that the alternative, nitrite-free processing concept maintained microbiological stability across all batches analysed. Samples were taken from finished products released for retail distribution; shelf-life testing at the end of the declared storage period was not part of this study.

### 5.5. Volatile N-Nitrosamines After Thermal Treatment

Volatile N-nitrosamines (VNAs) were quantified in additive-free, nitrite-free organic salami in unheated control samples and after thermal treatment at 200 °C for 12 min and 230 °C for 15 min. Across the entire analytical panel—N-nitrosodimethylamine (NDMA), N-nitrosodiethylamine (NDEA), N-nitrosodipropylamine (NDPA), N-nitrosodibutylamine (NDBA), N-nitrosomorpholine (NMOR), N-nitrosopiperidine (NPIP) and N-nitrosopyrrolidine (NPYR)—all unheated control samples showed concentrations below the analytical detection limit (LOD < 0.5 µg/kg) (Table 2), indicating the absence of pre-existing volatile nitrosamines in the finished product.

After heating at 200 °C for 12 min, VNA concentrations remained below the detection limit for all analytes, with no detectable increases compared with unheated controls (Table 2). In contrast, exposure to the high-temperature stress condition of 230 °C for 15 min resulted in small but measurable increases for selected compounds. Mean concentrations reached 0.8 ± 0.2 µg/kg for NDMA, 1.2 ± 0.3 µg/kg for NPIP, and 2.0 ± 0.4 µg/kg for NPYR, whereas NDEA, NDPA, NDBA and NMOR remained below the detection limit under all heating conditions (Table 2).

Statistical evaluation revealed that the increase observed for NDMA at 230 °C was not significant, whereas NPIP and NPYR showed significant increases compared with control samples and the 200 °C treatment (*p* < 0.05). Importantly, all analytes remained below the detection limit under conditions representative of typical domestic heating.

Overall, these results demonstrate that additive-free organic salami produced without nitrite curing salt does not form detectable volatile nitrosamines under realistic household heating conditions. Even under severe thermal stress, only low concentrations of selected cyclic nitrosamines were formed, remaining at toxicologically non-critical levels.

## 6. Discussion

### 6.1. Technological Concept, Process Robustness, and Microbiological Safety Without NPS

The results of this study applying the processing concept described here indicate that additive-free organic salami can be manufactured under industrial conditions without nitrite curing salt (NPS) while maintaining reliable process control and microbiological safety. This is technologically relevant because nitrite typically provides multiple functions in fermented sausages, including colour stabilisation, antioxidative effects, and antimicrobial contribution [1,2,3]. Omitting this hurdle requires that the remaining barriers are strengthened and precisely controlled.

Across two consecutive production years, the final product pH averaged 5.17 ± 0.18 across all batches with no significant year effect, indicating robust fermentation performance and consistent endpoint control. A pH in this range is typical for stable dry-fermented sausages and supports product stability when combined with sufficient dehydration [12]. Importantly, the pH profile must be interpreted in the context of additive-free processing: acidification acts as an early hurdle, limiting the growth potential of undesirable microbiota during fermentation and pre-ripening, while later stages increasingly rely on dehydration and reduced water activity to secure shelf stability. Microbiological results confirmed the effectiveness of this hurdle-based strategy. Hygiene indicators remained uniformly low, and relevant pathogens (*Listeria monocytogenes*, *Salmonella* spp., and STEC/VTEC targets) were not detected. These findings support the conclusion that, despite the absence of the antimicrobial contribution of nitrite, the combination of controlled fermentation, starter culture dominance, and drying is sufficient to ensure microbiological safety under industrial production conditions [12].

Residual nitrate and nitrite concentrations were low in both production years and showed no significant year effect. This observation is consistent with the nitrite-free production concept and reflects the combined influence of background nitrate/nitrite from raw materials and spices, metabolic conversion by starter cultures, and process-driven depletion during fermentation and ripening [1,2,7]. From a technological perspective, these findings are central, as they demonstrate that the alternative curing approach enables stable colour formation without elevated residual nitrite levels—an outcome that is particularly relevant for subsequent nitrosamine risk. From a mechanistic and process-technological perspective, these consistently low residual nitrate and nitrite concentrations further confirm the controlled nature of the alternative curing concept. In contrast to conventional nitrite curing, where nitrite is deliberately added in excess to ensure colour formation and antimicrobial action, the present approach relies on a quantitatively limited nitrate input and its biologically mediated conversion [2,3]. Nitrate derived from technological vegetable- and spice-based ingredients serves primarily as a transient precursor, which is enzymatically reduced by nitrate-reducing staphylococci and subsequently converted to nitric oxide during early fermentation and pre-ripening [1,11]. The low residual nitrite levels measured in the final products indicate that nitrite acts mainly as a short-lived intermediate rather than accumulating in the matrix, a condition regarded as technologically favourable from both a quality and safety perspective [1,7].

This controlled nitrate–nitrite–nitric oxide sequence is relevant for two reasons. First, it indicates that cured colour formation can proceed without the addition of nitrite curing salt; it should be noted, however, that colour was not measured instrumentally in the present study, so this interpretation rests on the residual nitrate and nitrite data and on routine visual assessment during production. Second, the limited availability of nitrosating agents at the end of ripening directly reduces the potential for nitrosamine formation during storage or subsequent thermal treatment, as previously described for fermented meat products with low nitrite availability [7,13]. Finally, the process monitoring concept likely contributed to the tight endpoint distributions observed across production years. Such monitoring is established practice in larger dry-sausage plants; its relevance here lies less in the monitoring itself than in the fact that the endpoint criteria had to compensate for the absence of nitrite. Standardised control of temperature, humidity, and airflow, together with batch-level tracking of drying progression (mass loss) and pH verification at defined process stages, provides an industrially feasible framework for reproducibility. While final water activity was targeted via defined dehydration, direct *aw* measurement would further strengthen process validation and facilitate comparability across calibres and seasonal conditions, particularly when transferring the concept to other production environments.

### 6.2. Nitrosamine Formation Under Domestic Heating and Relevance for Consumer Safety

The nitrosamine results demonstrate that volatile N-nitrosamines were not quantifiable in additive-free, nitrite-free organic salami in the unheated state and remained below the detection limit after heating at 200 °C for 12 min. A measurable increase occurred only under high-temperature stress at 230 °C for 15 min, where small amounts of NDMA, NPIP and NPYR were detected, while all other analytes remained below the detection limit. The ANOVA results confirmed that NPIP and NPYR increased significantly at 230 °C, whereas NDMA showed only a slight, non-significant increase. These findings indicate a limited nitrosation potential in the product matrix. In conventionally cured meats, nitrite provides the key precursor for nitrosating species; in the present salami, only trace residual nitrate and nitrite were present, limiting precursor availability and thereby reducing the potential for nitrosamine formation [1,3,7]. The absence of any increase at 200 °C suggests that typical domestic heating does not create relevant nitrosating conditions in this matrix. The minor formation of NPIP and NPYR under severe heat stress is consistent with prior observations that cyclic nitrosamines can be favoured at elevated temperatures in the presence of suitable amine precursors [13,34]. Under these conditions, localised surface dehydration and temperature gradients may promote reactions at the product surface, where nitrosating intermediates can form more readily than in the moist interior.

From a consumer safety perspective, the measured concentrations—even under extreme overheating—were low and close to the analytical detection limit. The key practical conclusion is therefore that additive-free, nitrite-free organic salami does not form relevant volatile nitrosamines under realistic household heating conditions, and that even severe overheating results only in minor increases at levels considered toxicologically non-critical. This outcome aligns with the overall process concept: low residual nitrite in the final product limits the substrate pool for nitrosation and contributes directly to the low VNA profile.

### 6.3. Methodological Considerations, Limitations, and Outlook

Several methodological aspects support the robustness of the conclusions. The GC–TEA approach is highly selective for volatile nitrosamines and is well suited to detect low-level changes under defined heating scenarios. Reporting results below the detection limit without numerical substitution avoids bias associated with artificial imputation of censored values. At the same time, the method is limited to volatile nitrosamines; non-volatile reaction products were outside the scope of this study. The dataset covers three independent production batches and two temperature–time regimens, providing a representative basis for consumer-level oven heating. Further work could expand the matrix of heating conditions (e.g., longer durations, lower temperatures, and additional cooking modes such as frying or grilling) to refine the boundary conditions for nitrosamine formation. In addition, integrating direct aw measurements into routine endpoint verification would strengthen the technological validation of the hurdle concept and improve transferability across product calibres and seasonal production variability [12].

Several further limitations should be acknowledged when interpreting these findings. The study was conducted with a single product formulation manufactured by one standardised process at one production facility. Although the industrial validation covered 20 batches across two consecutive production years, the results therefore apply to the investigated product and processing conditions rather than to nitrite-free fermented sausages in general. The analysis of volatile N-nitrosamines was based on three independent batches from a single production year, which further restricts the generalisability of the corresponding safety statements.

No conventional nitrite-cured control produced under identical processing conditions was included. While this does not affect the validity of the process data reported here, it limits the extent to which the technological consequences of omitting nitrite can be quantified, and a matched comparison is recommended for future work.

The hurdles applied in this concept—starter culture dominance, fermentation-driven acidification, pH reduction, dehydration and strict process control—acted simultaneously and were not varied independently. Their individual contributions to microbiological stability can therefore not be separated on the basis of the present data; the observed stability should be attributed to the combination of hurdles, with the reduction in water activity representing the decisive final barrier. Finally, instrumental colour measurement, lipid oxidation markers, enumeration of sulfite-reducing clostridia and formal sensory evaluation with a trained panel were outside the scope of this study. Product appearance and eating quality were assessed only qualitatively by the producer’s in-house expert panel as part of routine quality control, which judged the products to be typical of the category, with a cured colour that was slightly less intense than in nitrite-cured references but within the expected range. As these assessments were neither standardised nor conducted against a matched nitrite-cured control, no conclusions on colour stability, oxidative stability or consumer acceptability can be drawn from the present dataset. These parameters should be addressed in dedicated follow-up work.

Taken together, the combined results indicate that the process for industrial-scale production of additive-free organic salami without nitrite curing salt is feasible and yields products with stable fermentation behaviour, favourable microbiological status, low residual nitrate/nitrite levels, and a very low nitrosamine formation potential under realistic domestic heating.

## 7. Conclusions

### 7.1. Technological Feasibility and Product Safety Without Nitrite Curing Salt

This study provides evidence, based on the parameters determined here, that additive-free organic salami can be produced reliably at industrial scale without nitrite curing salt while maintaining microbiological safety and technological consistency. Across two consecutive production years, fermentation and ripening yielded stable final pH values (mean 5.17 ± 0.18) with no significant year effect. Residual nitrate and nitrite concentrations remained low within the lower two-digit mg/kg range, and no relevant pathogens—including *Listeria monocytogenes*, *Salmonella* spp., or Shiga toxin-producing *Escherichia coli*—were detected. Hygiene indicators were consistently low and complied with applicable guideline values.

These findings confirm that the omission of nitrite curing salt can be compensated by a precisely controlled, hurdle-based processing concept. Key elements include the use of functionally differentiated starter cultures, controlled acidification during early fermentation, and progressive dehydration during ripening, culminating in a low final water activity (overall mean 0.881; all batches ≤ 0.90) as the decisive barrier for long-term microbiological stability. Standardised control of temperature, relative humidity, airflow, and drying progression enabled reproducible processing outcomes across batches and production years. Collectively, these results provide a technological basis for the industrial production of the investigated clean-label, nitrite-free organic salami. Confirmation across further formulations, calibres and production sites would be required before extending these conclusions to nitrite-free fermented sausages more generally.

### 7.2. Nitrosamine Formation Under Domestic Heating and Consumer Relevance

In addition to technological feasibility and microbiological safety, the present study indicates a very low potential for volatile N-nitrosamine formation in the additive-free, nitrite-free organic salami investigated here, based on three independent production batches and two heating regimens. No volatile nitrosamines were detectable in unheated products or after heating under conditions representative of typical household preparation (200 °C for 12 min). Only under severe overheating conditions (230 °C for 15 min) were small increases observed for selected cyclic nitrosamines, namely N-nitrosopiperidine and N-nitrosopyrrolidine, while all other analytes remained below the analytical detection limit. Even under these stress conditions, measured concentrations were low and considered toxicologically non-critical. The favourable nitrosamine profile is directly linked to the alternative curing strategy employed, which results in low residual nitrite levels in the finished product and thus limits the availability of nitrosating precursors. From a consumer safety perspective, these findings indicate that additive-free, nitrite-free organic salami does not pose a relevant nitrosamine risk under realistic domestic heating conditions. Excessive overheating may induce minor increases, but these remain far below levels of health concern.

### 7.3. Final Considerations

Taken together, the results demonstrate that industrial-scale production of additive-free organic salami without nitrite curing salt is technologically feasible and yields products that combine microbiological safety, process robustness, and a very low nitrosamine formation potential. For the product and processing conditions investigated, the study provides a scientific and technological basis for the further development and industrial implementation of clean-label fermented meat products in the organic sector.

## Figures and Tables

**Table 1:** Microbiological results of additive-free organic salami (*n* = 20).

Parameter	Unit	Result
*Escherichia coli*	CFU/g	<10 in all samples
*Enterobacteriaceae*	CFU/g	<200 (10–200) in all samples
Coagulase-positive Staphylococci	CFU/g	<20 (1–20) in all samples
*Listeria monocytogenes* (quantitative enumeration)	CFU/g	n.d. in all samples
*Listeria monocytogenes* (qualitative detection in 25 g test portion)	CFU/g	n.d. in all samples
*Salmonella* spp. (qualitative detection in 25 g test portion)	CFU/g	n.d. in all samples
Shiga toxin-producing *Escherichia coli* (STEC/VTEC)—stx1 gene (PCR screening)	CFU/g	n.d. in all samples
Shiga toxin-producing *Escherichia coli* (STEC/VTEC)—stx2 gene (PCR screening)	CFU/g	n.d. in all samples

n.d. = not detected.

**Table 2:** Volatile N-nitrosamines (µg/kg) in additive-free organic salami before and after thermal treatment.

Compound	Control (Unheated)	200 °C/12 Min	230 °C/15 Min
NDMA (N-nitrosodimethylamine)	<0.5	<0.5	0.8 ± 0.2
NDEA (N-nitrosodiethylamine)	<0.5	<0.5	<0.5
NDPA (N-nitrosodipropylamine)	<0.5	<0.5	<0.5
NDBA (N-nitrosodibutylamine)	<0.5	<0.5	<0.5
NMOR (N-nitrosomorpholine)	<0.5	<0.5	<0.5
NPIP (N-nitrosopiperidine)	<0.5	<0.5	1.2 ± 0.3 *
NPYR (N-nitrosopyrrolidine)	<0.5	<0.5	2.0 ± 0.4 *

Values are mean ± SD (*n* = 3 independent batches). Values < 0.5 µg/kg represent concentrations below the analytical detection limit (LOD). * Significant increase compared with control and 200 °C treatment (*p* < 0.05).

## Data Availability

The data presented in this study are available on request from the corresponding author. The data are not publicly available because they originate from routine commercial production and laboratory records of the producing company.

## References

[1] Honikel K.-O. (2008). The use and control of nitrate and nitrite for the processing of meat products. Meat Sci..

[2] Sebranek J.G., Bacus J.N. (2007). Cured meat products without direct addition of nitrate or nitrite: What are the issues?. Meat Sci..

[3] Sindelar J.J., Milkowski A.L. (2011). Sodium Nitrite in Processed Meat and Poultry Meats: A Review of Curing and Examining the Risk/Benefit of Its Use.

[4] IARC (1978). Some N-Nitroso Compounds.

[5] IARC (2010). Ingested Nitrate and Nitrite, and Cyanobacterial Peptide Toxins.

[6] Jakszyn P., González C.A. (2006). Nitrosamine and related food intake and gastric and oesophageal cancer risk: A systematic review. World J. Gastroenterol..

[7] Bedale W., Sindelar J.J., Milkowski A.L. (2016). Dietary nitrate and nitrite: Benefits, risks, and evolving perceptions. Meat Sci..

[8] Asioli D., Aschemann-Witzel J., Caputo V., Vecchio R., Annunziata A., Næs T., Varela P. (2017). Making sense of the “clean label” trend: A review of consumer food choice behaviour and discussion of industry implications. Food Res. Int..

[9] European Union (2018). Regulation (EU) 2018/848 of the European Parliament and of the Council of 30 May 2018 on organic production and labelling of organic products. Off. J. Eur. Union.

[10] Alahakoon A.U., Jayasena D.D., Ramachandra S., Jo C. (2015). Alternatives to nitrite in processed meat: Up to date. Trends Food Sci. Technol..

[11] Toldrá F. (2015). Handbook of Fermented Meat and Poultry.

[12] Leistner L. (2000). Basic aspects of food preservation by hurdle technology. Int. J. Food Microbiol..

[13] Drabik-Markiewicz G., De Mey E., Bekaert K., Smet S., Bille J., Vandekerckhove P., Paelinck H. (2011). Influence of putrescine, cadaverine, spermidine or spermine on the formation of N-nitrosamines in heated cured pork meat. Food Chem..

[14] Leistner L., Gould G.W. (2002). Hurdle Technologies: Combination Treatments for Food Stability, Safety and Quality.

[15] Hammes W.P., Hertel C. (1998). New developments in meat starter cultures. Meat Sci..

[16] Leroy F., Verluyten J., De Vuyst L. (2006). Functional meat starter cultures for improved sausage fermentation. Int. J. Food Microbiol..

[17] Ravyts F., De Vuyst L., Leroy F. (2012). Bacterial diversity and functionalities in food fermentations. Eng. Life Sci..

[18] Skibsted L.H. (2011). Nitric oxide and quality and safety of muscle based foods. Nitric Oxide.

[19] Bourafai-Aziez A., Jacob D., Charpentier G., Cassin E., Rousselot G., Moing A., Deborde C. (2022). Development, Validation, and Use of 1H-NMR Spectroscopy for Evaluating the Quality of Acerola-Based Food Supplements and Quantifying Ascorbic Acid. Molecules.

[20] Bühler S. (2023). Production of Organic Salami without Additives: Technological and Quality Aspects. Doctoral Dissertation.

[21] (1999). Meat and Meat Products—Measurement of pH—Reference Method.

[22] (1998). Foodstuffs—Determination of Nitrate and/or Nitrite Content—Part 7: Continuous Flow (CFA) Method for the Determination of Nitrate after Cadmium Reduction.

[23] (2017). General Requirements for the Competence of Testing and Calibration Laboratories.

[24] BVL (2026). Bestimmung von flüchtigen N-Nitrosaminen in Lebensmitteln [Determination of volatile N-nitrosamines in foodstuffs]; Method L 00.00-17. Amtliche Sammlung von Untersuchungsverfahren nach § 64 LFGB (ASU).

[25] (2013). Microbiology of the food Chain—Enumeration of Microorganisms—Surface Plating at 30 °C.

[26] (2001). Microbiology of Food and Animal Feeding Stuffs—Enumeration of β-Glucuronidase-Positive Escherichia coli.

[27] (2021). Microbiology of the Food Chain—Enumeration of Coagulase-Positive Staphylococci.

[28] (2017). Microbiology of the Food Chain—Enumeration of Enterobacteriaceae.

[29] (2017). Microbiology of the Food Chain—Detection of Salmonella spp. (Amendment 1:2020).

[30] (2017). Microbiology of the Food Chain—Detection of Listeria monocytogenes.

[31] (2017). Microbiology of the Food Chain—Enumeration of Listeria monocytogenes.

[32] DGHM (2017). Mikrobiologische Leit- und Warnwerte für Lebensmittel (Kategorie: Rohwürste/Fermentwürste).

[33] WHO (2003). Nitrate and Nitrite in Drinking-Water.

[34] Drabik-Markiewicz G., De Mey E., Peeters M.-C., Bekaert K., Smet S., De Smet S., Paelinck H. (2009). Role of proline and hydroxyproline in N-nitrosamine formation during heating of cured meat. Food Chem..

